# Patients’ ideas, concerns, expectations and satisfaction in primary health care – a questionnaire study of patients and health care professionals’ perspectives

**DOI:** 10.1080/02813432.2019.1684430

**Published:** 2019-11-14

**Authors:** Joel Freilich, Eivor Wiking, Gunnar H Nilsson, Christina Olsson

**Affiliations:** a Roslags Näsby Health Care Center, Stockholm County Council, Stockholm, Sweden;; b Mörby Academic Primary Health Care Center, Stockholm County Council, Stockholm, Sweden;; c Department of Learning, Informatics, Management and Ethics (LIME), Karolinska Institutet, Stockholm, Sweden;; d Department of Neurobiology, Care Sciences and Society (NVS), Karolinska Institutet, Stockholm, Sweden

**Keywords:** Primary health care, patient centeredness, general practitioner, district nurse, physiotherapist

## Abstract

**Objective:** Explore the perceptions of patients and health care professionals about patients’ ideas, concerns, expectations (ICE), and satisfaction in consultations with general practitioners (GPs), district nurses (DNs) and physiotherapists (PTs).

**Design:** Cross-sectional questionnaire study of participants in planned consultations.

**Setting:** Five primary health care centers and two rehabilitation centers in Stockholm, Sweden.

**Subjects:** Pairs of patients and GPs (*n* = 156), patients and DNs (*n* = 73), and patients and PTs (*n* = 69).

**Main outcome measures:** Multiple-choice questions about patients’ ICE and satisfaction.

**Results:** Approximately 75% of patients and GPs reported that patients’ thoughts and explanations about their symptoms emerged during the consultation. For patient-DN pairs, the figure was 60%, and for patient-PT pairs, 80%. A majority of patients reported not having concerns and anxiety about the investigation/treatment, whereas health care professionals thought patients were more concerned. One-third of patients consulting GPs and PTs expected to receive a reason/explanation for their symptoms. Figures were lower for the DNs. About 70% of patients were satisfied with the consultation.

**Conclusions:** Most patients expressed their ideas, a minority had concerns, and a minority expected an explanation of their illness. Patients and health care professionals rated patient satisfaction high, but health care professionals tended to believe patients were less satisfied than patients reported they were.

**Key points**
- Patient surveys show that important aspects of patient-centeredness remain weak in Swedish primary health care; for example, shared decision-making.
- In this study of planned consultations, few patients expected to receive an explanation of their symptoms, but most were satisfied with the consultation.
- Health care professionals thought patients’ experiences were more negative than they were.
- This discrepancy was observed in responses to questions about patients’ concerns, expectations and satisfaction.

## Introduction

A patient-centered consultation starts with eliciting the patient’s perspective. Most patients have a particular agenda, which often includes ideas about the cause of the consultation [1]. In many cases, they also have an explanation of why they do not feel well [1,2]. Earlier studies have shown that patients do not always express their entire agenda in consultations, which can lead to misunderstandings and poor outcomes, such as unwanted prescriptions and non-adherence to treatment [3]. It is therefore important that health care professionals explore patients’ ideas, concerns and expectations (ICE) early in the consultation [3–5].

In patient-centered consultations, health care professionals aim to share understanding and decision-making with patients [6]. This involves empathy with and respect for the patient. Patient-centeredness may lead to increased patient satisfaction [7,8], better adherence to treatment [9,10], less need for investigations and fewer prescriptions [11,12], fewer referrals [13], better health outcomes [10,14] and less health care utilization [13,15].

Swedish health care policy calls for patient-centered care [16]. However, an analysis of Swedish National Patient Survey data from the mid-2000s showed deficiencies in involving Primary health care (PHC) patients in planning their care [17]. Moreover, a 2017 survey found that patients older than 65 years in Sweden were receiving less information and shared less in decision-making than previously and in Europe as a whole [18]. It is therefore important to investigate how care can become more patient-centered for patients in Sweden.

PHC serves as the foundation of health care systems, and many patient consultations take place there. Most studies have focused on patients’ experiences of consultations [1–4,7], but it is also important to gain insight into the experiences of health care professionals, since achieving concordance is a central part of patient-centered consultations. Few previous studies have examined patients and health care professionals’ experiences of patients’ ICE and satisfaction in the same consultations [19].

The aim of this study was to explore the perceptions of patients and health care professionals about patients’ ICE and satisfaction in consultations with general practitioners (GPs), district nurses (DNs) and physiotherapists (PTs), the largest groups of health care professionals in PHC.

## Method

### Design

This study is a cross-sectional questionnaire study of patients and health care professionals who took part in planned consultations.

### Setting

The study was conducted at five PHC centers and two rehabilitation centers in northeast Stockholm from 1 February 2015 to 31 July 2015. Ten of the 28 PHC centers in the northeast area of Stockholm were invited to participate in the study. Invitations were made by telephone and via in-person visits from one of the researchers. The research group was familiar with the centers in the area, and the 10 PHC centers were chosen because their staff situation was stable and they had shown previous interest in research. Six of the centers agreed to participate in the study, but one dropped out after a few months because of the heavy workload at the center. One of the PHC centers had all three categories of professionals, and five had GPs and DNs but no PTs. Two rehabilitation centers only had PTs. Only fully trained specialist physicians in family medicine were included in the study.

In northeastern Stockholm, socioeconomic status is generally high, and Swedish is the most commonly used language. The population of the three municipalities represented in this study has a higher educational level than most other areas in Stockholm and Sweden as a whole.

### Questionnaires

Two questionnaires were developed: one for patients, which asked about their experiences, and one for health care professionals, which asked about patients’ experiences. The questions were based on items in questionnaires used in earlier studies of patient-centeredness [20–25]. Because none of the previously existing questionnaires addressed all the items we wanted to include in this study, we developed the study-specific questionnaires. A research group that included GPs, district nurses, physiotherapists, and senior researchers revised, translated and adapted the questions to Swedish PHC. The questions addressed background factors and ideas (questions 2 and 3), concerns (questions 5–8), expectations (questions 9–11 and 13–14) and satisfaction (questions 16–20) (Tables 2 and 3). Response alternatives were ‘yes’, ‘partly’, ‘no’ and ‘I don’t know’. Questions 1, 4, 12 and 15 were open-ended and were not included in this study but will be part of a forthcoming qualitative study. The questionnaires were tested on pairs of patients and health care professionals (3 GPs and 3 patients, 3 DNs and 3 patients, and 3 PTs and 3 patients). The patients and health care professionals deemed the questions understandable, so no changes were made to them.

### Sampling and ethics

All managers and participating professionals provided oral informed consent before the study started. The receptionists also gave patients oral and written information about the study prior to inclusion, highlighting the voluntary and anonymous nature of participation. The receptionists at the centers consecutively invited Swedish-speaking adult patients to participate. They were to invite as many patients as possible during the study period who were booked for consultations with GPs, DNs and PTs and to keep track of the number of patients who declined to participate. Only patients attending planned consultations were invited, not those attending acute care consultations. It was not possible to include acute care consultations because such consultations are too short for both patients and professionals to have time to provide reflections about patients’ ICE.

After obtaining patients’ oral informed consent, the receptionist provided them with two anonymous questionnaires with matching codes: one for the health care professional and one for the patient. The codes enabled the researchers to match the responses from the same consultation. Immediately following the consultation, patients and health care professionals were to separately complete their questionnaires and return them to the receptionist. The coded anonymous surveys were returned by the patients to the receptionist, either handed in or left in a sealed box. The receptionist kept the completed questionnaires in sealed boxes until the researcher collected them. The participating centers were of varied size and recruited different numbers of participants. The length of time questionnaires were distributed and collected also varied by center.

The study was approved by the Regional Ethics Review Board in Stockholm, Sweden, Dnr 2014/1851-31.

## Results

### Study population

A total of 724 questionnaires were distributed, and 641 were returned by patients and health care professionals and collected from the centers by one of the researchers. These questionnaires included responses from 156 pairs of patients and GPs, 73 pairs of patients and DNs, and 69 pairs of patients and PTs (a total of 298 consultations) (Table 1). Thirty-five patients who were invited declined to participate or returned a blank questionnaire (7 who consulted GPs, 19 who consulted DNs, and 9 who consulted PTs). The majority of respondents were women. Most patients who consulted GPs and DNs were ≥50 years.

**Table 1.** Gender, age distribution and number of participants in 298 consultations with general practitioners (GP), district nurses (DN) and physiotherapists (PT).

	GP (*n* = 156)						DN (*n* = 73)						PT (*n* = 69)					
	Patient			GP			Patient			DN			Patient			PT		
	W	M	U	W	M	U	W	M	U	W	M	U	W	M	U	W	M	U
Age (years)	98	55	3	88	66	2	34	33	6	60	10	3	47	22	0	55	14	0
20–49	40		5	56		6	5		7	20		11	19		2	41		1
50–70	59			94			21			42			26			27		
>70	52						40						22					

M: men; W: women; U: unanswered/unknown gender or age.

Of the health care professionals, GPs had the most equal gender distribution. On average, PTs were younger than GPs and DNs.

The most common causes of consultations with GPs were musculoskeletal, circulatory, and psychological problems; with DNs, were related to wound dressing, blood pressure measurement, and medical supplies; and with PTs, were for musculoskeletal problems.

This study analyzed data at the group level to investigate patterns in responses from patients and health care professionals.

### Ideas, concerns and expectations

Three-quarters of the patients and a corresponding percentage of GPs reported that patients’ thoughts and explanations about their symptoms emerged during the consultation (Table 2, question 2). Around 60% of patients and DNs reported that the patients presented their thoughts and explanations. The highest figure was observed for PT consultations, where over 80% of patients felt that their thoughts and explanations emerged during the consultation. Approximately 70% of patients and health care professionals reported that patients’ questions about health were answered (question 3).

A minority of patients reported that they had concerns about the cause of their illness (7–14%; Table 2, question 5) or investigation/treatment (4–7%, question 6). On the other hand, 25% of GPs perceived that their patients had concerns about the cause of their illness; in DNs and PTs the numbers were lower, but a total of 44% of DNs and 46% of PTs partly agreed that the patient had such concerns (question 5). In 11% to 33% of consultations, patients reported that they had presented their concerns (question 7).

**Table 2.** Question number and content, and distribution in percentages of answers from 298 patient consultations with general practitioners (GP), district nurses (DN) and physiotherapists (PT) regarding related ideas, concerns and expectations.

		GP (*n* = 156)								DN (*n* = 73)								PT (*n* = 69)							
		Patients				GP				Patients				DN				Patients				PT			
Question area, number and content		Yes	Partly yes	No	D/U	Yes	Partly yes	No	D/U	Yes	Partly yes	No	D/U	Yes	Partly yes	No	D/U	Yes	Partly yes	No	D/U	Yes	Partly yes	No	D/U
*Ideas*																									
2	If patients’ thoughts and explanations about their symptoms emerged during the consultation	75.6	8.3	9.0	7.1	73.1	22.4	2.6	1.9	63.0	5.5	9.6	21.9	60.3	20.5	4.1	15.1	84.0	8.7	5.9	1.4	68.1	26.1	4.4	1.4
3	If patients' questions about health were answered	70.0	17.9	3.8	8.3	76.3	19.9	1.9	1.9	68.5	8.2	5.5	17.8	74.0	16.4	6.9	2.7	79.7	17.4	2.9	–	69.6	27.5	–	29
5	If patients thought there was something frightening that might cause their symptoms	7.1	19.2	62.8	10.9	25.0	26.9	44.3	3.8	6.8	6.8	69.9	16.5	5.5	43.8	42.5	8.2	14.5	17.4	58.0	10.1	17.4	46.4	36.2	–
*Concerns*																									
6	If patients had concerns and anxiety regarding investigation/treatment	7.1	14.7	72.4	5.8	9.0	18.6	71.8	0.6	5.5	6.8	80.9	6.8	2.7	28.8	54.8	13.7	4.3	11.6	79.7	4.3	8.7	27.5	56.5	7.3
7	If patients' concerns were presented	23.1	16.7	41.0	19.2	17.3	18.6	63.5	0.6	10.9	13.7	56.2	19.2	4.1	21.9	68.5	5.5	33.3	17.4	37.7	11.6	14.5	31.9	52.2	1.4
8	If patients had other concerns	10.3	11.5	68.6	9.6	10.3	11.5	68.6	9.6	12.2	11.0	59.0	17.8	15.1	31.5	50.7	2.7	11.6	10.1	69.6	8.7	23.2	30.4	43.5	2.9
*Expectations*																									
9	If patients had any expectations about receiving a reason/an explanation for their symptoms	31.4	16.7	40.4	11.5	40.4	30.8	28.2	0.6	12.3	12.3	59.0	16.4	20.5	17.8	52.1	9.6	31.2	31.2	26.1	10.1	44.9	29.0	23.2	2.9
10	If patients felt they were respected and taken seriously	95.5	–	–	4.5	95.6	3.8	0.6	0	87.6	5.5	1.4	5.5	90.4	1.4	2.7	5.5	98.6	–	–	1.4	97.1	2.9	–	–
11	If patients' expectations for the consultation were fulfilled	87.8	8.3	1.3	2.6	76.9	19.2	2.6	1.3	83.6	5.5	2.7	8.2	68.5	20.5	–	11.0	88.4	5.8	1.4	4.3	71.0	24.7	2.9	1.4
13	If patients received help with what they expected	84.0	9.6	0.6	5.8	80.8	17.9	1.3	–	76.7	2.7	1.4	19.2	74.0	16.4	1.4	8.2	85.5	11.6	–	2.9	65.2	33.4	1.4	–
14	If patients thought there was anything that should have been done at the consultation but was missed	3.8	2.6	84.6	9.0	30.8	9.0	59.6	0.6	1.4	1.4	79.5	17.7	15.1	5.5	76.7	2.7	1.4	4.3	88.4	5.8	20.3	13.0	66.7	–

D/U: Don’t know/unanswered.

About a third of patients consulting GPs (31%) and PTs (32%) expected to receive an explanation for their symptoms. The figures were lower for DNs (12%; Table 2, question 9). A high percentage of patients felt their expectations for the consultation had been fulfilled (84–88%, question 11) and that they were respected and taken seriously (88–98%, question 10). These figures are in line with those of the health care professionals. Few patients felt something was missed during their consultation, whereas a higher percentage of health care professionals perceived that patients felt this way (question 14).

### Questions about satisfaction

The majority of patients reported that they were satisfied with the way the health care professional behaved towards them, with the information and emotional support they received, and with shared decision-making (74–94%, Table 3, questions 16–19). The highest proportion of satisfied patients was observed in PT consultations. A lower percentage of all groups of health care professionals than patients reported that patients were satisfied with the consultation (questions 16–20).

**Table 3.** Question number and content, and distribution in percentages of answers from 298 patient consultations with general practitioners (GP), district nurses (DN) and physiotherapists (PT) related satisfaction.

		GP (*n* = 156)								DN (*n* = 73)								PT (*n* = 69)							
		Patients				GP				Patients				DN				Patients				PT			
Question number and content		Yes	Partly yes	Partly no/No	D/U	Yes	Partly yes	Partly no/No	D/U	Yes	Partly yes	Partly no/No	D/U	Yes	Partly yes	Partly no/No	D/U	Yes	Partly yes	Partly no/No	D/U	Yes	Partly yes	Partly no/No	D/U
*Satisfaction*																									
16	If patients were satisfied with how they were behaved towards during the consultation	91.7	3.8	–	4.5	66.6	32.1	1.3	–	84.9	1.4	0	13.7	72.6	20.6	2.7	4.1	94.2	2.9	–	2.9	82.6	16.0	–	1.4
17	If patients felt they received enough information	80.8	12.2	1.3	5.7	66.0	32.8	0.6	0.6	75.3	9.6	1.4	13.7	54.8	38.3	1.4	5.5	88.4	8.7	–	2.9	65.3	31.9	1.4	1.4
18	If patients felt involved in decisions about investigations and treatment	84.6	9.0	1.3	5.1	66.6	32.8	0.6	–	74.0	8.2	–	1.,8	61.7	30.1	2.7	5.5	87.0	4.4	1.4	7.2	59.4	33.3	5.9	1.4
19	If patients felt they received sufficient emotional support during the consultation	87.8	5.8	1.3	5.1	68.6	29.5	0.6	1.3	78.1	4.1	1.4	1.4	75.3	16.5	1.4	6.8	89.9	5.8	–	4.3	63.8	31.9	1.4	2.9
20	If patients were satisfied with the consultations a whole	86.0	8.3	0.6	5.1	66.0	32.8	0.6	0.6	84.9	6.9	2.7	5.5	75.3	17.9	–	6.8	87.0	10.1	–	2.9	56.5	39.2	1.4	2.9

D/U: Don’t know/unanswered.

## Discussion

This study explored patients’ ICE and satisfaction as expressed by patients and health care professionals who had taken part in the same PHC consultation. We found that most patients had expressed their ideas, but fewer had presented their concerns. A relatively low number expected to receive an explanation for their symptoms, but most felt their overall expectations had been met. Health care professionals tended to believe patients were less satisfied than patients reported they were.

### Ideas

The number of patients who expressed their own ideas (their own thoughts and explanations) about their illness was higher than in an earlier study in which doctors failed to elicit about half of patients’ problems [26]. The relatively low numbers who expressed their ideas in DN consultations could stem from the purpose of the consultations (e.g. blood pressure measurement, wound dressing). Fewer patients may have felt it was relevant to express their own ideas about their illness during such consultations. The highest numbers of patients who presented their ideas were found in the group who consulted PTs. This could be because patients often consult PTs for musculoskeletal problems, and the causes of such problems can be difficult to determine. Moreover, patients typically consult PTs repeatedly for the same cause, providing patients with several opportunities to tell their story.

### Concerns

It is possible that the relatively low percentage of patients in our study (23%) and in a previous study of GP trainees (35%) [11] who presented concerns about their illness during consultations may originate in the planned nature of the consultations in both studies. That is, patients may have brought up their concerns in earlier consultations. The same may be true of patients consulting PTs (33%), whereas the corresponding results for DNs (12%) may have to do with the nature of the consultation, as described previously.

### Expectations

Overall, patients felt their expectations had been met. However, only about a third of patients consulting GPs and PTs expected to receive an explanation of the cause of their illness. Perhaps this is because we studied planned (mainly follow-up) consultations, so patients may already have received such an explanation. The lower proportion of patients consulting DNs (12%) who expected to receive an explanation for their illness may reflect the non-complex nature of many of these consultations.

### Satisfaction

Overall, a high percentage of patients and health care professionals reported that patients were satisfied with the consultation as a whole. Earlier studies that focused on patients’ perspectives have found that contextual factors (e.g. shorter waiting times and care continuity) [27–30], patient characteristics (e.g. age and functional status) [31,32], and factors related to the consultation are associated with patient satisfaction. Consultation-related factors include receiving an explanation of the illness [21,31–33], receiving emotional support [31], and feeling respected by and experiencing good communication with the health care professional [34].

Many patients in the current study (74–87%) were also satisfied with opportunities for shared decision-making regardless of which kind of health care professional they consulted. This contrasts with the results of a previous study that found that patients of nurses were more satisfied with shared medication-related decision-making than patients of GPs or pharmacist prescribers [35]. It also seems inconsistent with the findings of the 2017 survey that showed that older patients in Sweden shared less in decision-making than older patients in other countries in Europe [18]. One potential explanation for the seeming inconstancy is that the survey covered all forms of health care, not only PHC. Continuity of care delivery and the more natural environment in PHC might facilitate shared decision-making.

We hypothesize that the relatively high proportion of patients who were satisfied with shared decision-making may be related to the high socioeconomic status of our study area. Previous studies indicate that high socioeconomic status is linked to a preference for a more active role in shared decision-making [32,36]. Thus, patients in our study area may have taken more initiative to discuss decisions with the health care professionals. However, this is just a hypothesis, and further study would be needed to test it.

Our main finding was the discrepancy between patients’ reported satisfaction and health care professionals’ perceptions of patient satisfaction. We observed this finding in all three professions. There could be many reasons for the discrepancy. For instance, health care professionals may have wanted to bring up more topics than time permitted, or they may not have had the opportunity to check that the patient’s needs had been met. Previous studies show that PHC professionals experience a great deal of job stress [37–39], and misperception of patients’ experiences might be a preventable source of such stress.

### Strengths and weaknesses

The focus on both patients’ and health care professionals’ perspectives of the same consultation is unusual, as is the inclusion of consultations with three different kinds of primary health care professionals. Another strength is the fairly large number of respondents. Moreover, the inclusion of multiple health care centers may make the results more generalizable. On the other hand, all participating centers were located in a part of Stockholm characterized by higher socioeconomic status than the country as whole, which negatively affects generalizability.

The main limitation of the study was the use of an unvalidated questionnaire, which affects the validity and reliability of the results. It is not clear whether study participants grasped the concepts of ICE that lay behind the questions, and differing understanding of the questions could have affected responses. The questionnaire is in need of further testing, which should include tests of validity and reliability, as well as qualitative investigation into how respondents interpret the questions. Furthermore, it is difficult to compare the results of the current study with those of previous studies because of differences in methodology and interpretations of the concept of ICE.

Furthermore, the questionnaires were completed immediately after the consultation, so patients did not have the opportunity to reflect on their experiences. Another limitation is that we do not know whether this was patients’ first consultation with the health care professional or whether it was the first time they had consulted a PHC professional about that specific illness. It is also possible that professionals’ encounters with the patients could have been affected by the fact that the professionals knew they were participating in the study. Finally, we did not perform any power calculation, since the study was descriptive and the distribution of collected data was not well known.

## Conclusions

The main finding of the study was the discrepancy between patients’ self-reported concerns, expectations, and satisfaction, and health care professionals’ perceptions of these factors. The finding was observed in all three professions. Professionals might wish to achieve more than they are able to in a single consultation, or they may forget to ask patients about their perceptions of the consultation. We suggest that health care professionals’ misperception of patient satisfaction might be a source of stress, so further studies seem warranted on this topic. Future studies could also investigate ICE and satisfaction in acute consultations to see whether patterns are similar and explore health care professionals’ satisfaction with consultations.
